# [Retracted] Interaction of YAP1 and mTOR promotes bladder cancer progression

**DOI:** 10.3892/ijo.2024.5638

**Published:** 2024-03-19

**Authors:** Mingxi Xu, Meng Gu, Juan Zhou, Jun Da, Zhong Wang

Int J Oncol 56: 232-242, 2020; DOI: 10.3892/ijo.2019.4922

Following the publication of the above article, a concerned reader drew to the Editor's attention that certain of the immunohistochemical data shown in Fig. 1C on p. 236, and immunofluorescence data featured in Figs. 2G and 5G on p. 237 and 239 respectively, were strikingly similar to data that had appeared in other articles written by different authors at different research institutes which had already been published.

In view of the fact that certain of the data in the above article had already been published at the time of the paper's submission, the Editor of *International Journal of Oncology* has decided that this paper should be retracted from the publication. After having been in contact with the authors, they accepted the decision to retract the paper. The Editor apologizes to the readership for any inconvenience caused.

